# Threat to Cedar, *Cedrela odorata*, Plantations in Vietnam by the Weevil, *Aclees* sp.

**DOI:** 10.1673/031.010.19201

**Published:** 2010-11-04

**Authors:** Pham Quang Thu, Dao Ngoc Quang, Bernard Dell

**Affiliations:** ^1^ Forest Science Institute of Vietnam, Hanoi, Vietnam; ^2^ Institute of Sustainable Ecosystems, Murdoch University, Perth, Australia

**Keywords:** Curculionidae, Hylobina, Spanish cedar, tree death, wood borer

## Abstract

The recent decline and death of young cedar, *Cedrela odorata* L. (Sapindales: Meliaceae), plantations in Vietnam is caused by *Aclees* sp. (Coleoptera: Curculionidae), a wood-boring brown weevil. A field study was undertaken in three-year-old plantations in two districts in Thanh Hoa province in August 2008. Trees were heavily impacted by the weevil, *Aclees*; the infestation level (P) ranged from 80 to 100% and the average damage index (R) ranged from 1.8 to 2.8. Observations over one year enabled the life history to be determined. Eggs were laid (February to March, September to November) inside the bark from the base of the trunk up to 60 cm in height. Larvae formed extensive feeding tunnels in the inner bark and sap wood. Pupation occurred in feeding tunnels or pupal chambers in the sapwood. Adults emerged twice a year, February to March and August to October. It is concluded that *Aclees* is a threat to *C. odorata* plantations in tropical regions of the world, and quarantine measures should be implemented to reduce the risk of spread.

## Introduction

Native to the tropical region of America, the Spanish cedar *Cedrela odorata* L. (Sapindales: Meliaceae) is naturally distributed from northern Mexico through Central America to Argentina and throughout the Caribbean islands (Pennington 1981). *C. odorata* is commercially the most important and widely distributed species in the genus. The wood is highly valued for furniture and other uses. It is a deciduous tree that can reach 35 m in height and 60 cm in diameter at breast height (Rosca 2003). Seeds from 3 provenances (Villa Hermosa, Gusman, and Campeche) were brought to Vietnam in 1986 (Loc 2008). The provenances were planted in the North of Vietnam in 1988, reaching a diameter of 48–64 cm after 14 years. In 2005 this species was recommended for planting in three ecological zones: Northeast (Thai Nguyen province; average rainfall 1620 mm), Central North (Thanh Hoa province; average rainfall 1835 mm), and High Land (Dak Lak and Gia Lai provinces; average rainfall 1900 – 2270 mm) regions. In 2008 seed from four more provenances, three from Costa Rica (Perez Zeledóón, Guanacaste and Sarapiqui) and one from Honduras, were imported for planting in five provinces: Quang Ninh, Nghe An, Yen Bai, Kon Tum, and Binh Phuoc. Each location measured 6 hectares (Dong 2008).

In the past two years the brown weevil, *Aclees* sp. (Coleoptera: Curculionidae), has become a severe pest of young *C. odorata* plantations in Central-North Vietnam and threatens the viability of this new plantation industry. Larvae cause most of the damage as they feed and bore into the inner bark and sapwood causing damaged trees to die.

The purpose of this paper is to describe the life cycle of *Aclees* and the damage it causes in order to alert the plantation forestry sector of this new emerging pest.

## Materials and Methods

### Assessment of damage

**Figure 1.**
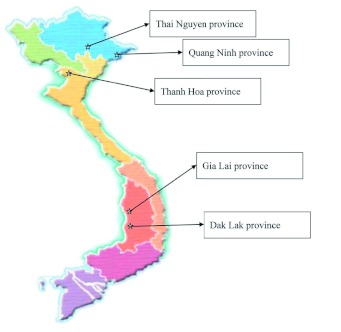
Map of Vietnam showing the five provinces where experimetal plantations of *Cedrela odorata* have been established. The brown weevil is present in Thanh Hoa province.

Field observations were carried out during August 2008 at three-year-old plantations in the Thanh Hoa province (Figure 1) of central-North Vietnam, where there was local concern for the decline of *C. odorata* in trial plantings. Thanh Hoa province has a tropical monsoon climate with four distinct seasons. The mean annual rainfall is 1600–2300 mm, there are 90–130 rain days per year, the relative humidity is high (85–87%), and the mean average temperature is 23–24°° C. Six plots, measuring 400 m^2^, were randomly laid out in the Thuong Xuan and Ba Thuoc districts of the Thanh Hoa province (three plots in each location). All 40 plants in each plot (ca. 10–15 cm in diameter at breast height and 3–4 m height) were checked for *Aclees* attack and damage was scored against the following four categories:

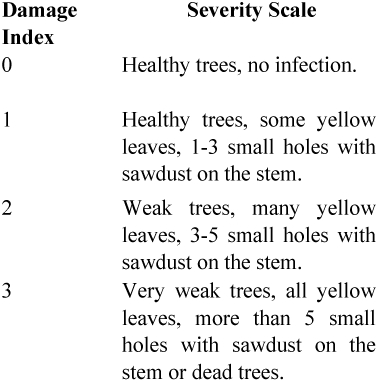


The damage incidence (percentage of trees affected) in each plot was calculated as follows:

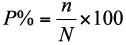


Where *n* = the number of trees attacked by *Aclees* and *N* = number of trees in a plot. The average damage index in each plot was calculated as follows:

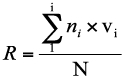


Where R = the average damage index; ni = the number of trees infected at damage index I; v_i_ = the damage index at level I; and N = the number of trees assessed. Based on the average damage index, the damage severity level was ranked as follows:

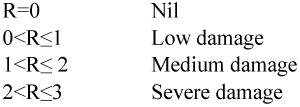


### *Aclees* biology and identification

*Aclees* larvae were reared in the laboratory in Hanoi using logs taken from the plantations until adults were obtained. Additional observations were obtained seasonally in the field. Adult body length was measured along the midline from the anterior of the eye to the apex of the elytra, and width was measured across the dorsal plate at the widest point. Identification to genus was based on keys in Morimoto (1982) and Thompson (1992). Sixteen adult specimens of *Aclees* were deposited in Forest Science Institute of Vietnam (FSIV) with numbers coded from 200001 to 200018. The cytochrome oxidase 1 High quality figures are available online. gene region was sequenced using mtDNA extracted from the legs of two adult specimens (200017, 200018) and compared to data on Genbank in order to confirm the subfamily for *Aclees*. Protocols were performed as described by Laffin et al. (2005) except that air-dried specimens were used. The primers used were Jerry, Mila, and Pat (Simon et al. 1994).

## Results

### Causal agent, distribution, and damage

In all cases, *Aclees* was associated with tree decline and death. On the basis of adult morphology it was identified to *Aclees* and is probably an undescribed species. Cytochrome oxidase 1 analysis confirmed that *Aclees* is within the Hylobina, being 91% similar to *Hylobius abietis*.

**Figure 2.**
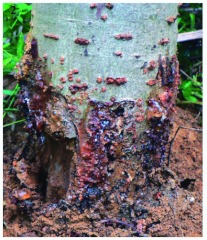
Three year old *Cedrela odorata* tree with oozing resin and saw dust at the base of the trunk.

**Figure 3.**
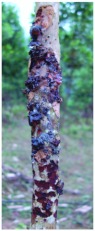
One year old *Cedrela odorata* stem showing damage to stem 1 m above the ground caused by *Aclees* sp. High quality figures are available online.

**Figure 4.**
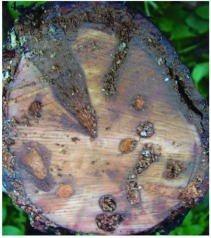
Three year old *Cedrela odorata* stem damaged by larvae of *Aclees* sp.

*Aclees* was first reported in North Central Vietnam in 2006. According to the surveys of the Forest Plant Protection Research Division (FSIV) undertaken in 2006, 2007, and 2008, *Aclees* occurs throughout Thanh Hoa province. Infestation by *Aclees* (Figures 2, 3, 4) was associated with three types of damage: leaf chlorosis, growth reduction, and increased susceptibility to wood decay organisms.

In Thanh Hoa province, the infestation level (P%) of *Aclees* in all six plots was very high, ranging from 80 to 92.5% and 95 to 100% in Ba Thuoc and Thuong Xuan districts, respectively (Table 1). Trees in all plots were heavily impacted by the brown weevil with the average damage index (R) ranging from 1.8 to 2.8 (Table 1).

## Description

### Larvae

**Figure 5.**
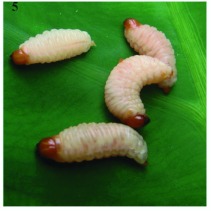
*Aclees* sp. larvae. High quality figures are available online.

Larvae are C-shaped grubs, white to greyish-white with a yellow-brown head. They lack legs and have few large hairs. The first instar larvae are about 2 mm long and the last instar larvae reach a length of up to 12 mm (Figure 5).

### Pupae

**Figure 6.**
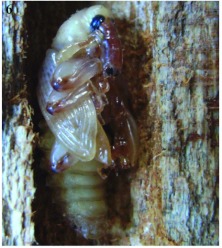
*Aclees* sp. pre-pupa inside *C. odorata* stem.

Pupae somewhat resemble the adults except the wings are not fully developed, they have large setae on the thorax and abdomen with a couple of long setae on the last abdominal segment, and they are white to yellowish in colour (Figure 6). Morphological and anatomical features are illustrated in Figure 8.

**Table 1.**
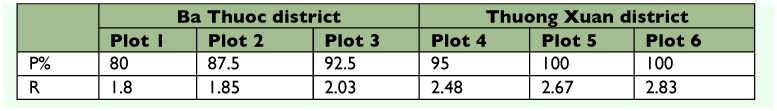
Damage incidence and average damage index of the brown weevil in two districts in Thanh Hoa province, Vietnam.

**Figure 7.**
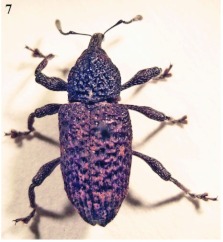
*Aclees* sp. adult female. High quality figures are available online.

### Adult

Adults of *Aclees* vary considerably in size, and the female (Figure 7) is usually larger than the male, with lengths between 11.5 – 13.5 mm and 10.5 – 12.5 mm, respectively; and widths between 4.2 – 4.5 mm and 4.0 – 4.3 mm, respectively. The body is arched with a long stout beak. The femora of all legs have two sharp spurs near the end. The colour varies from light brown to nearly black, with rows of pits or punctures on the dorsal surface. The thorax and elytra are covered with small scales. The eggs are oval and white when first deposited. They measure 0.7 – 1.0 mm in length and 0.5 – 0.7 mm in width.

### Life cycle

Adults begin to lay eggs anywhere between several weeks to a few months after emergence. The eggs are deposited singly inside the bark at the base of the tree extending up to 60 cm above the ground. Hatching occurs after an incubation period of two weeks or more depending on the temperature. After hatching, larvae bore in the bark and sapwood, the bark turns brown with the oozed sap/resin and associated sawdust (Figure 2), and trees appear stressed with yellow and sometimes wilted leaves. The first three to four instars feed principally on the inner phloem and cambial tissues, and the last two instars proceed to feed on sapwood where they make extensive tunnels (Figure 4). Larvae take up to four months to become fully grown. Pupation occurs in the feeding tunnels or in pupal chambers in the sapwood. Pupation lasts from several weeks to one month depending on the ambient temperature. Adults remain in the tunnel for a few days after eclosion, and the wood lining the pupal chambers becomes discoloured (Figure 4).

**Figure 8.**
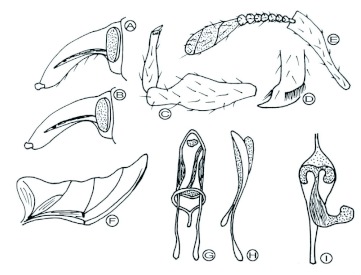
Details of *Aclees* sp. A) head and rostrum, male; B) head and rostrum, female; C) fore leg; D) apex of hind tibia; E) antenna; F) hind wing; G) aedeagus, dorsal; H) aedeagus, lateral; I) female genital organ, dorsal. High quality figures are available online.

Overwintering adult *Aclees* appear in the spring and begin egg laying for the first generation in February and March. Adults from the second generation emerge over a three month period from August to October, with the most concentrated emergence in September. Adults of this generation lay their eggs on infested trees into undamaged bark above the last infestation zone. Adults feed on the green bark and juvenile leaves of *C. odorata* causing stunted growth when grazing intensity is high. Adults feed at night from the outer margin of the leaf inwards, creating characteristic crescent-shaped notches, and these notches can be used as an early indicator of potential larvae inside the stem near the base of young seedlings.

## Discussion

This is the first report of borer damage to *C. odorata* in Vietnam. Early symptoms include premature leaf chlorosis (similar to nitrogen deficiency) (Webb et al. 2001), unthrifty crowns, and copious resin secretion. Unpublished observations from the Thanh Hoa province indicate that severely impacted trees die 1–2 years after the initial tunnel damage from larvae of *Aclees.* Trees aged 1–3 years are most susceptible to *Aclees* damage in Vietnam. Surveys undertaken by the Forest Plant Protection Research Division (FSIV) in August 2008 revealed that all *C. odorata* provenance trials planted in August 2005 in the Ba Thuoc and Thuong Xuan districts of the Thanh Hoa province were decimated three years after planting. So far, only young stands have been severely damaged. It is unknown whether older trees are susceptible. There did not appear to be any predisposing factors to *Aclees* attack. Stands were established on upland sites with good drainage, and no symptoms of nutrient deficiencies were visible in other tree species in the area. Also, there was no evidence of previous damage from other pests and disease organisms.

The extent of damage to young stands by *Aclees* sp. is of great concern as it threatens the viability of new plantations in SE Asia. So far the insect is only known to be a problem in Vietnam, but could quickly spread into neighboring countries. Care should be taken to ensure that biosecurity measures are put into place to prevent the introduction of this pest to other continents, particularly the Americas. All plantations of *Cedrela* should be checked for infestations from this borer. This can easily be done by looking for small holes, usually ≤?6 mm in diameter, that have sawdust falling from them or that are oozing sap. Sawdust on the tree trunk or ground is also a useful indicator. The presence of clean holes, without sawdust, indicates the adults have already emerged. Holes with sawdust indicate the immature borer is still feeding inside the tree.

Further work is in progress to identify the species of this weevil. The genus *Aclees* currently contains about 28 species principally distributed on the western rim of the Pacific, from Japan to Papua New Guinea. Several are well known pests of *Ficus* (Verghese et al. 2003) and one species has become a pest on *Ficus carica* in Italy resulting in tree death due to larval damage to the wood in the collar region (Ciampolini et al. 2007). The Hylobiina contains some serious wood-boring pests of trees in the northern Hemisphere, including species of *Hylobius* associated with conifers.

Search for wood boring weevils in secondary forests adjacent to plantations failed to reveal the presence of *Aclees*. Native Meliaceae is widely distributed in forests in SE Asia and wood borers are unknown. *Melia azesdarach* is planted in hedge rows and is commonly used in upland swidden agriculture. No infested trees were encountered in roadside surveys. Presently, the natural distribution of the *Aclees* is unknown. *Aclees* could have been introduced into Vietnam with the importation of seed. However, this is unlikely as *Aclees* has not been reported as damaging to *C. odorata* in the regions from where seed was obtained. It is more likely that *Aclees* is associated with populations of some Meliaceae in the Asian region, where there is great diversity of genera (Hua Peng et al. 2008). It is unlikely that *Aclees* has changed its host from *Ficus* to *Cedrela*.

Since *Aclees* kills young trees it is more destructive than *Hypsipyla robusta* (Lepidoptera: Pyralidae) which has been the main concern (Cunningham et al. 2005) in establishing commercial plantations of the Swietenioideae subfamily of the Meliaceae in the region. The only recorded Coleoptera damaging *C. odorata* trees or seedlings are *Xyleborus biseriatus* (Coleoptera: Scolytidae) and *Oncideres albomarginata* (Coleoptera: Cerambycidae) in South America (Garrido and Briceno 1997; Briceno et al. 2004).

So far, no provenances planted in Vietnam are resistant to *Aclees*. Until control measures have been successfully established for *Aclees* further planting of *C. odorata* in Vietnam is not recommended. It is possible that natural resistance exists as *C. odorata* has a wide geographical distribution from mesic to dry habitats and considerable variation occurs in morphological and physiological traits (Navarro et al. 2002).

## Conclusions

The viability of *Cedrela odorata* plantations in Vietnam are under threat from a new stem borer that is described here for the first time. Whether *Aclees* can infest other commercially important genera in the Swietenioideae subfamily of the Meliaceae is unknown. Quarantine measures should be implemented to prevent further spread of the pest in SE Asia.
